# Comparison of EEG propagation speeds under emotional stimuli on smartphone between the different anxiety states

**DOI:** 10.3389/fnhum.2014.01006

**Published:** 2014-12-10

**Authors:** Tetsuya Asakawa, Ayumi Muramatsu, Takuto Hayashi, Tatsuya Urata, Masato Taya, Yuko Mizuno-Matsumoto

**Affiliations:** ^1^Department of Physiology and Biological Information, Dokkyo Medical UniversityTochigi, Japan; ^2^Graduate School of Applied Informatics, University of HyogoHyogo, Japan; ^3^Department of Medical Engineering, Aino UniversityOsaka, Japan; ^4^Faculty of The Physical Education, Osaka University of Health and Sports ScienceOsaka, Japan; ^5^KDDI R & D Laboratories Inc.Saitama, Japan

**Keywords:** EEG, smartphone, emotional stimuli, anxiety states, propagation speed

## Abstract

The current study evaluated the effect of different anxiety states on information processing as measured by an electroencephalography (EEG) using emotional stimuli on a smartphone. Twenty-three healthy subjects were assessed for their anxiety states using The State Trait Anxiety Inventory (STAI) and divided into two groups: low anxiety (I, II) or high anxiety (III and IV, V). An EEG was performed while the participant was presented with emotionally laden audiovisual stimuli (resting, pleasant, and unpleasant sessions) and emotionally laden sentence stimuli (pleasant sentence, unpleasant sentence sessions) and EEG data was analyzed using propagation speed analysis. The propagation speed of the low anxiety group at the medial coronal for resting stimuli for all time segments was higher than those of high anxiety group. The low anxiety group propagation speeds at the medial sagittal for unpleasant stimuli in the 0–30 and 60–150 s time frames were higher than those of high anxiety group. The propagation speeds at 150 s for all stimuli in the low anxiety group were significantly higher than the correspondent propagation speeds of the high anxiety group. These events suggest that neural information processes concerning emotional stimuli differ based on current anxiety state.

## Introduction

The Ministry of Internal Affairs and Communications of Japan has reported that the market penetration of mobile communication terminals such as cellular phones has increased from 0.3% in 1989 to 98.0% in 2012 (Ministry of Health, Labor and Welfare, 2012) and it continues to grow. Recently, smartphones have become popular as mobile communication terminals, acting not only as telephones but as information and communication terminals. The use of smartphones has been reported to produce new relationships over age, distance, and status (Ministry of Health, Labor and Welfare, 2012). Use of the smartphone has spread rapidly in recent years. Out of the world population, the penetration rate of smartphones has reached 70%. For many, the smartphone has become a necessity in private work and in daily life (ITmedia, 2008; Nikkei BPnet, 2008). On the other hand, smartphones are thought to be a cause of physical and mental stress, and overdependence or overuse of smartphones has been reported to cause depression, sleep disorders, and other symptoms of stress (Thomée et al., 2011). Stress induced by smartphone use has not been clarified and may be categorized as a new type. Biological responses to emotional stress may be different from responses to emotional stress and may cause an as yet undetermined condition (Krause et al., 1998).

The current study is based on emotional stress stimulation using audiovisual stimuli which have been used in previous studies (Hayashi et al., 2009; Mizuno-Matsumoto et al., 2011). Researchers examined the effect of emotional stimuli on a specific emotional stress reaction within a living organism using emotional stimuli on a smartphone. Although it is possible to display images, video, voice, and words using audio-visual stimuli to stimulate an emotional stress response, the primary feature of smartphones is logging in to e-mail and Web pages that communicate with friends and family, such as Facebook. Therefore, using the smartphone differs from conventional emotional stimuli because others may provide stimuli in displayed text and images.

Part of the current study included performing propagation speed analysis between neighboring electrodes. Results were also evaluated using power spectral analysis in EEG (Kamo et al., 2013). There are two possible methods which can be used for analysis between electrodes: coherence analysis and phase analysis. Propagation speed analysis is the primary analysis used in the current study which evolved from phase analysis. It is considered that high propagation speeds correlate with fast information processing. In previous studies, information propagation analysis has not been used for the evaluation of EEGs (electroencephalography) using emotional stimuli and differentiating between differences in anxiety state. The effect of anxiety states on information processing as measured using propagation speed analysis has not been previously studied.

The purpose of the current study is to evaluate the relationship between different anxiety states and neural information processing using emotional stimuli on a smartphone.

## Materials and methods

### Participants

The subjects included 23 healthy adults (9 males and 14 females) aged 22–45 years old (mean ages 25.5 ± 1.2 years). Written informed consent was obtained from all subjects before the start of the experiments. They had no history of neurological or psychiatric illness. The Ethical Committee of the University of Hyogo approved this investigation and informed consent was obtained according to the Declaration of Helsinki.

### Psychological test

Prior to the start of experiments, psychological tests were conducted to assess the anxiety conditions of the subjects. The State Trait Anxiety Inventory (STAI), a psychological inventory based on a 4-point Likert scale and consisting of 40 questions on a self-report basis was used to assess subjects' anxiety levels (Julian, 2011). The STAI measures two types of anxiety: state anxiety, or anxiety about an event, and trait anxiety, or anxiety level as a personal characteristic. Higher scores are positively correlated with higher levels of anxiety (Hidano et al., 2000; Ueno, 2003).

In this study, all participants were assessed for the presence of anxiety states using the trait anxiety measurement of the STAI. They were categorized into two categories: psychosomatically healthy, comprising subjects with scores of I and II, and psychosomatically ill, comprising subjects with scores of III, IV, and V. According to the results of the STAI, subjects were divided into two groups. Subjects with scores I and II were classified as the “low anxiety group” and those with scores III, IV, and V as the “high anxiety group.” Then the emotional stimuli-related cerebral activities of the two groups were compared.

### EEG experiment

After a brief instruction about the physiological test and the attachment of electrodes, subjects were placed in a shielded room (band cut filter of 0.5–500 MHz, attenuation of 40 dB) in a sitting position.

As an objective physiological evaluation, an EEG was performed for each subject. Electrooculogram (EOG) was recorded for the removal of eye-movement artifacts. The Ag-AgCl EEG electrodes were placed on 16 sites (Fp1, Fp2, F3, F4, C3, C4, P3, P4, O1, O2, F7, F8, T3, T4, T5, and T6) according to the International 10–20 System, which is recognized as a standard in cranial landmarks. The linked earlobes (Aav) were adopted as a reference. During recording, impedances of all electrodes were kept below 10 kΩ. The EEG data was digitized at a sampling rate of 500 Hz. **Figure 2** shows a subject's position during the sessions.

### Experiment protocol

**Figure 1:**
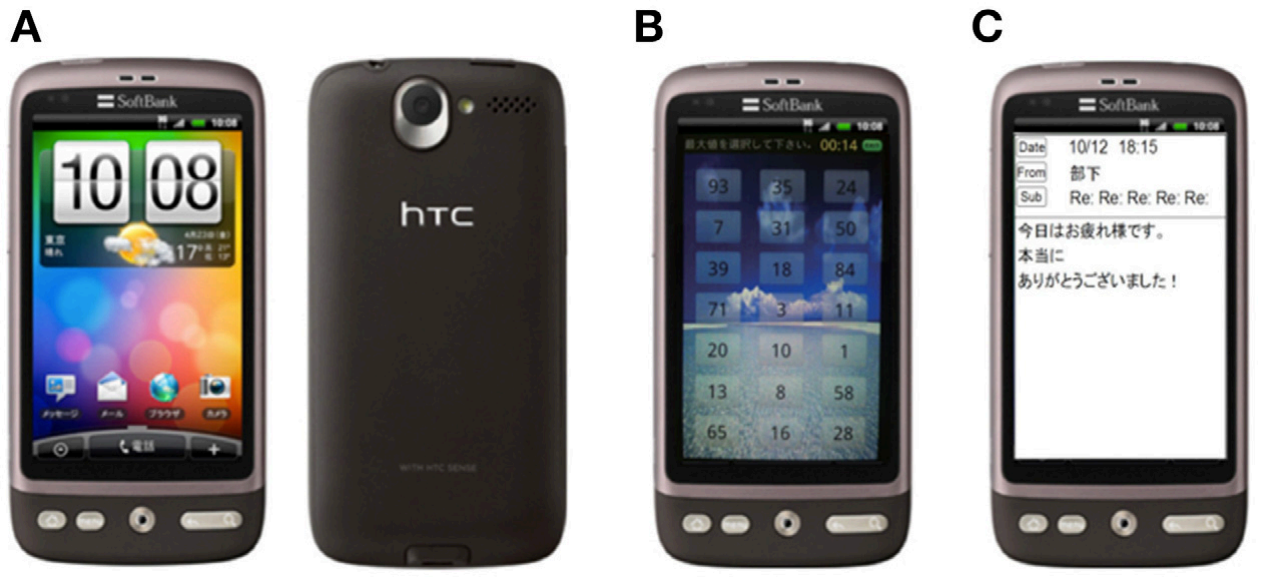
**The smartphone using experimental design. (A)** Desire (HTC Co. Ltd.), **(B)** emotional audio-visual stimuli (resting stimuli), **(C)** emotional sentence stimuli (pleasant sentence stimuli).

Each experiment consisted of a total of five sessions presenting emotional audio-visual and sentence stimuli using a smartphone. We performed Desire (HTC Co. Ltd.) of smartphone (see Figure 1A). Before use, the SIM card of the smartphone used in this study was unplugged so as not to cause artifacts.

Two different sessions using audio-visual stimuli was used: pleasant and unpleasant. Similarly, sentence stimuli were separated into pleasant and unpleasant sessions. The fifth condition was a rest session using a neutral audiovisual stimulus. The subjects were to follow the instructions that were displayed on the smartphone. As shown in Figure 1B, the screen showed random numbers during the audio-visual stimuli; the subjects selected the maximum or minimum numbers. Emotional stimuli sessions provided audio-visual stimuli of four types every 10 s for 40 s during the stimulation. Figure 1C shows an example of e-mail on a smartphone. At the same time, audio stimuli, sound effects, were presented.

Participants viewed relaxing pictures such as landscapes in the resting session, funny pictures such as animals in the pleasant session, and terror pictures such as horror movies in the unpleasant session. In the emotional sentence stimuli sessions, participants viewed funny sentences found in e-mail as pleasant sentence stimuli, and anxiety-provoking sentences from e-mail as the unpleasant sentence stimuli.

**Figure 2:**
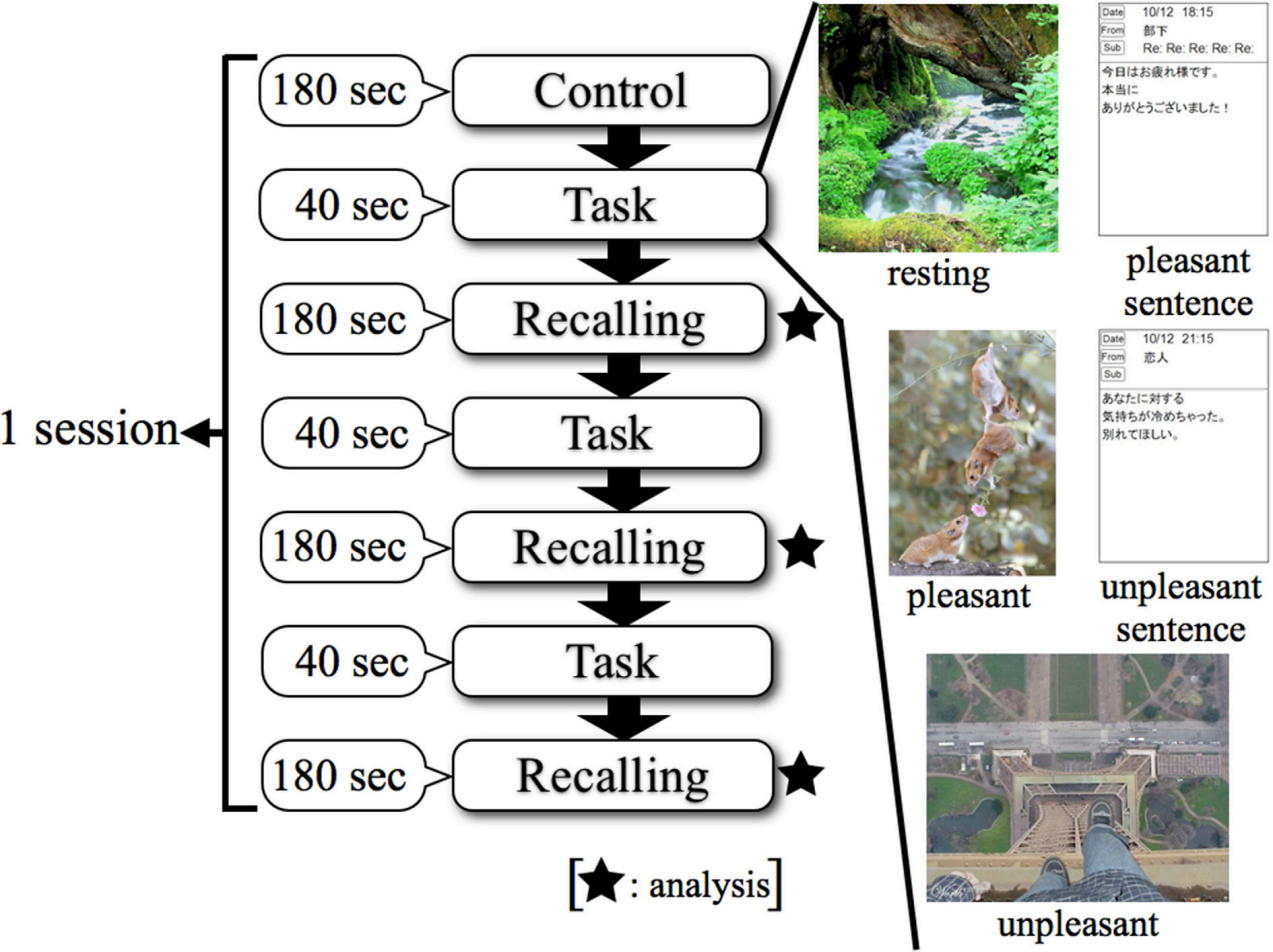
**Experimental protocol using smartphone in EEG**. Control: no-load state with eyes closed, Task: watching stimuli, Recalling: recalling the contents of stimuli with eyes closed.

**Figure 3:**
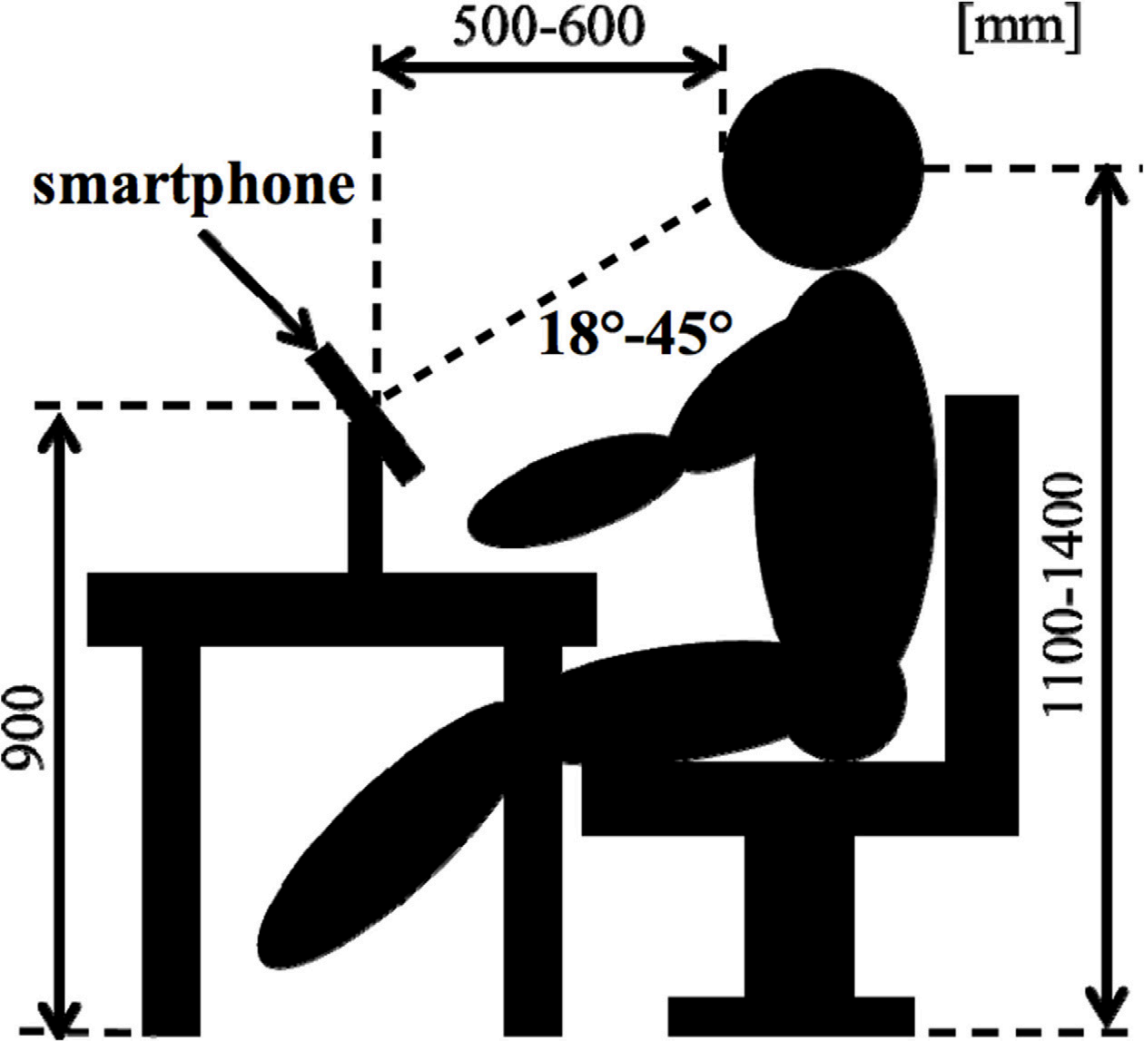
**Position of a subject during the tests**.

Figure 2 shows that the procedure for a session consisted of three steps. The first step was “Control,” in which the subject kept a quiescent state with eyes closed for 180 s; this phase was defined as a no-load state (Control). After the no-load state, the second step was “Task,” in which the subject watched stimuli for 40 s. The third step of a session was “Recalling,” in which the subject recalled the contents of the emotionally stimuli with eyes closed for 180 s. The second and third steps were performed three times consecutively. Thereafter, this series was performed in two replicates. This protocol was applied to each stimulus as 1 session, and a total of 5 sessions were performed which included resting, pleasant, and unpleasant stimuli. EEG measurements were performed continuously for 5 sessions. EEG results were analyzed in the “Recalling” phases. This study was designed based on experimental designs of previous research (Hayashi et al., 2009; Mizuno-Matsumoto et al., 2010; Miyagawa et al., 2013). Figure 3 shows a subject's position during the sessions.

### Analysis

#### Propagation speed analysis between neighboring regions

Artifacts were removed from the EEG data with an IIR band-pass filter of 2–50 Hz; large artifacts were manually removed from the analysis periods.

Propagation speed analysis between the brain regions was conducted to investigate the functional relationship between regions in the EEG data. Our in-house programs in MATLAB ver.7.7 were used for analysis.

The analysis epoch was 150 out of 180 s for recall. The number of points for analysis was 2048 and the frequency resolution was 0.488 Hz. The propagation speed for 4.1 s per 1 analysis period was calculated in 23 combinations between neighboring brain regions.

Furthermore, the 150 s per task were divided into 5 time windows (30 s per window), and propagation speeds for each time window were calculated between each pair of regions. We performed propagation speed in the α (8.0–14.0 Hz) band. Propagation speeds were calculated for each of the 5 kinds of tasks: resting, pleasant, unpleasant, pleasant sentence, and unpleasant sentence stimuli.

#### The definition of propagation analysis

The definition of propagation analysis is as below (Saiwaki et al., 1997; Mizuno-Matsumoto et al., 2000a). The Propagation analysis is based on the coherence and phase analyses. This expression used on cross-correlation ***C_xy_***(***u**ptau*), cross-spectral **S_xy_**(**ω**), real part ***K_xy_***(**ω**) and imaginary part ***Q_xy_***(**ω**). The cross-correlation ***C_xy_***(**τ**) of two time-series signals x(t) and y(t) was defined by the following equation. In previous research, propagation speed has been used for analysis of abnormal EEG epileptic activity. It was able to capture propagation of abnormal EEG activity to localize brain lesions (Mizuno-Matsumoto et al., 2000b). Overall, propagation speed analysis is basically used to measure the propagation of postsynaptic potentials between electrodes and their abnormalities.

$$C_{xy}\left(\tau \right)=\underset{T\to \infty }{lim}\frac{1}{2T}\int_{-T}^{T}x\left(t\right)y\left(t+\tau \right)dt$$

Here, τ was showed time interval. We performed the FFT of cross-correlation function, and required the cross-spectral ***S_xy_***(**ω**) was defined by the following equation.

$$S_{xy}\left(\omega \right)=\int_{-\infty }^{\infty }C_{xy}\left(\tau \right)e^{-j\omega \tau }d\tau$$

ω was showed angular frequency. ***S_xy_***(**ω**)divided into real part ***K_xy_***(**ω**) and imaginary part ***Q*_xy_**(**ω**). The phase by angular frequency ω between two signals was defined by the following equation.

$$\theta_{xy}=tan^{-\text{1}}\left\{\frac{Q_{xy}\left(\omega \right)}{K_{xy}\left(\omega \right)}\right\}\left(-\pi <\theta_{xy}<\pi \right)$$

Furthermore, time lag of frequency component ω was defined by the following equation.

$$\tau \left(\omega \right)=\frac{\theta_{xy}\left(\omega \right)}{\omega }$$

The propagation speed *v_p,q_* by distance *r_p,q_* between two electrodes p and q was defined by the following equation.

$$v_{p,q}\left[m/s\right]=\frac{r_{p,q}\left[mm\right]}{\tau \left(\omega \right)\left[ms\right]}$$

#### Statistical analysis

**Figure 4:**
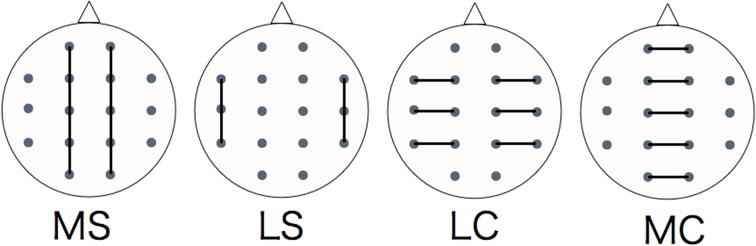
**Evaluating regions of propagation speed**. MS, medial sagittal; LS, lateral sagittal; LC, lateral coronal; MC, medial coronal.

We divided the analysis of propagation speeds between neighboring regions into four regions: medial sagittal, lateral sagittal, lateral coronal, and medial coronal (see Figure 4). Statistical analysis was used to measure the propagation speed between neighboring regions of each subject. The values between each region for each stimulus, time course, and group were compared using a Two-Way repeated-measures analysis of variance (ANOVA) with the Bonferroni correction for multiple comparisons. IBM SPSS Statistics ver. 21 was used for statistical analysis.

## Results

### Psychological tests

Using the STAI, 7 subjects had a score of I and 6 subjects had a score of II. Four subjects were categorized as score III, 5 subjects scored as IV, and 1 subject had score V. Therefore, we divided the subjects into two groups split at score II. Thus, 13 subjects were categorized as Low anxiety group, and 10 subjects were categorized as High anxiety group, respectively (see Table 1).

**Table 1:** **The number of the subjects and STAI**.

	**STAI (score)**				
	**I**	**II**	**III**	**IV**	**V**
Subject	7	6	4	5	1
Group	Low anxiety group		High anxiety group		

*Thirteen subjects were categorized as Low anxiety group, and 10 subjects were categorized as High anxiety group, respectively*.

### Propagation speed

**Figure 5:**
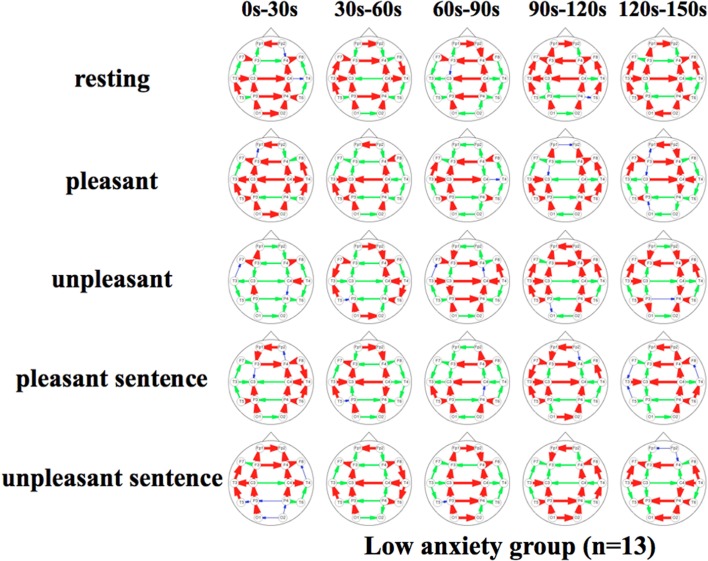
**Propagation speeds between each region in each stimulus of time series**. Drawing propagation speeds between each region in each stimulus of time series in Low anxiety group. And the thickness and color of lines displayed represent the coherence value. Bold red lines show a high level; green moderate-thickness lines show an intermediate level, and blue thin lines represent a low level.

**Figure 6:**
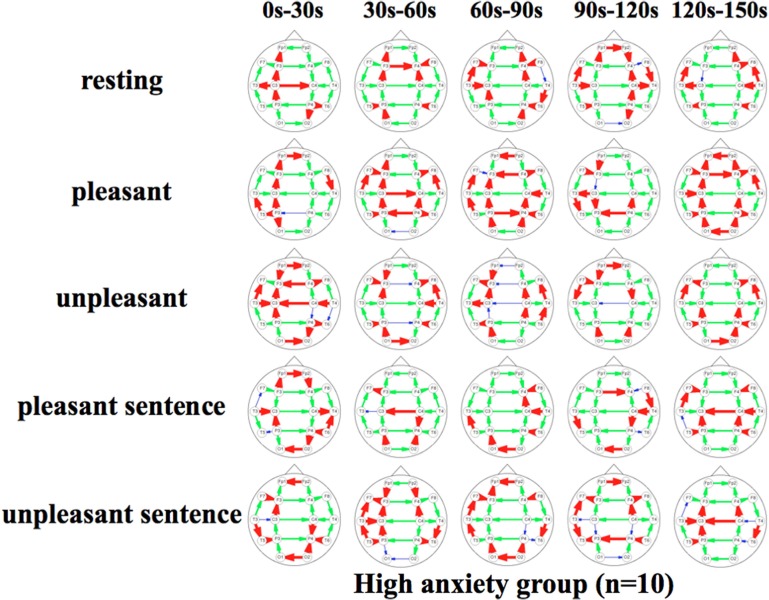
**Propagation speeds between each region in each stimulus of time series**. Drawing propagation speeds between each region in each stimulus of time series in High anxiety group. And the thickness and color of lines displayed represent the coherence value. Bold red lines show a high level; green moderate-thickness lines show an intermediate level, and blue thin lines represent a low level.

Figures 5, 6 show a graphical representation of propagation speeds between each region in each stimulus of time series in Low anxiety group and High anxiety group, respectively. The thickness and color of the lines displayed represent the propagation speed. Bold red lines show a high level (greater than or equal to 0.5 [m/s] and less than 25 [m/s]); green moderate-thickness lines show an intermediate level (greater than or equal to 0.05 [m/s] and less than 0.5 [m/s]), and blue thin lines represent a low level (more than 0 [m/s] and less than 0.05 [m/s]).

The results show that propagation speeds in the inter-hemisphere (Fp1-Fp2, F3-F4, C3-C4, P3-P4, O1-O2) of the low anxiety group were fast propagation speeds from 0 to 150 s in resting stimuli; from 0 to 90 and 120–150 s in pleasant stimuli; or 30–150 s in unpleasant stimuli' 0–150 s in pleasant sentence stimuli; and 0–150 s in unpleasant sentence stimuli. The same goes for both hemispheres of directions. Notably, all stimuli were propagated in a sagittal direction in the low anxiety group. The propagation speeds in the inter-hemisphere of the high anxiety group from 0 to 30 s in unpleasant and unpleasant sentence stimuli were faster than 30–150 s in those.

### Propagation speeds statically analysis

**Figure 7:**
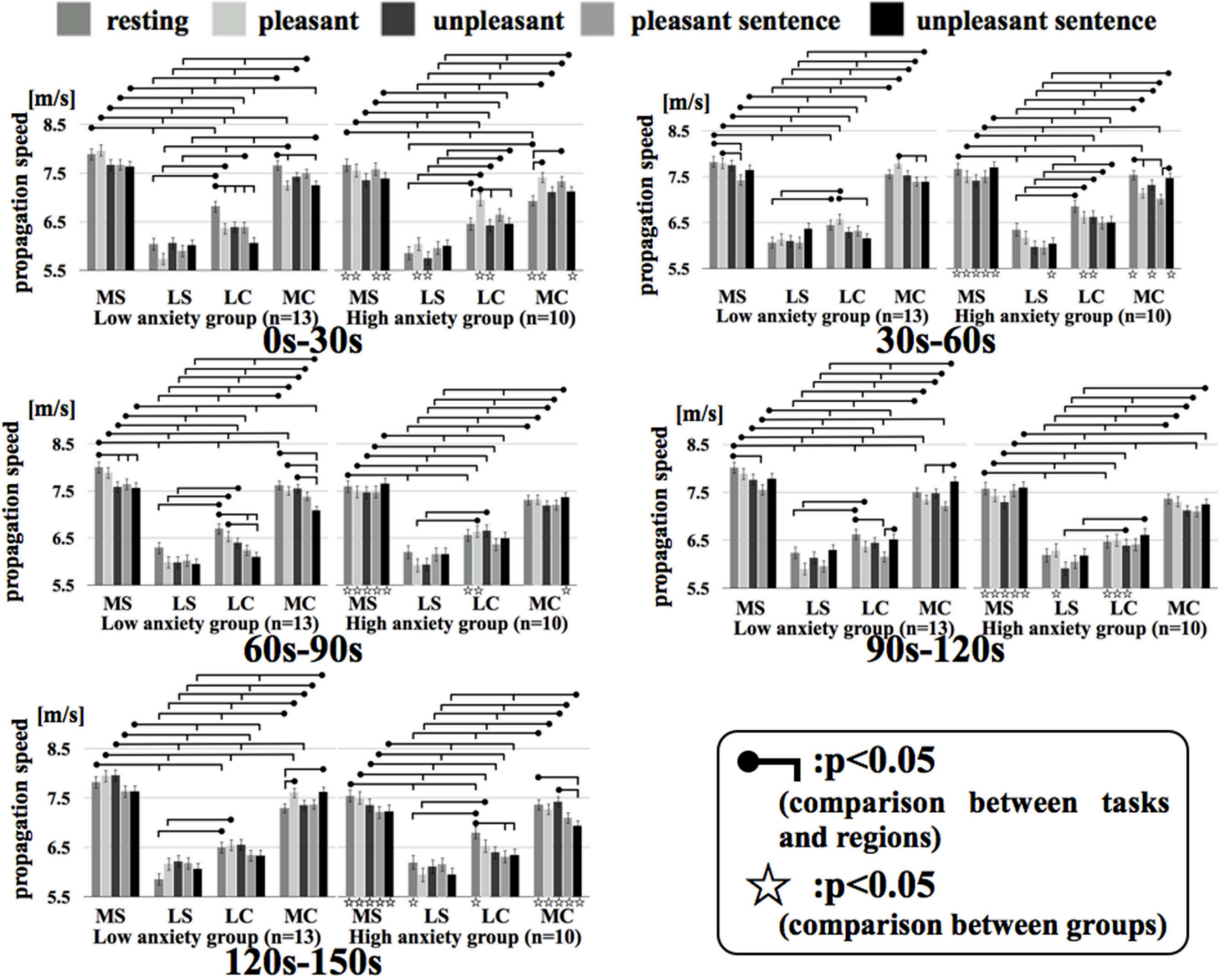
**Regional propagation speed in each time**. MS, medial sagittal; LS, lateral sagittal; LC, lateral coronal; MC, medial coronal. Two-Way factorial ANOVA for the propagation speed in all time. The vertical axis shows propagation speed and the horizontal axis shows each region examined in each task.

Figure 7 shows the results of a Two-Way factorial ANOVA for the propagation speed in all time intervals. The vertical axis shows propagation speed and the horizontal axis shows each region examined in each task.

In comparing the two groups, the propagation speeds in the medial coronal region for the low anxiety group were significantly higher than that in the high anxiety group (*p* < 0.05) during resting and unpleasant sentence stimuli of 0–30 s. The high anxiety group showed significantly higher propagation speeds in the medial coronal region for resting and unpleasant stimuli of 120–150 s than that in Low anxiety group (*p* < 0.05). Apart from the 0–30 s interval for unpleasant stimuli and the 60–90 s interval for unpleasant stimuli, the propagation speeds for resting, pleasant, and unpleasant stimuli in all time intervals for the medial sagittal region were significantly higher in the low anxiety group than that in High anxiety group (*p* < 0.05).

In comparison between regions, apart from the 0–30 s interval for resting stimuli and the 90–120 s interval for unpleasant stimuli in the high anxiety group between lateral coronal and medial coronal (*p* < 0.05), the propagation speed of the medial sagittal and medial coronal regions was significantly higher than the lateral sagittal and lateral coronal regions in both groups in all time intervals.

## Discussion

The present study was designed to evaluate information processing through EEG propagation speed analysis in the α (8.0–14.0 Hz) band for emotional stress stimuli presented on a smartphone. The difference in neural information processing based on the anxiety state of the subject was then extracted.

### Propagation analysis in the present study

This study calculated propagation speeds between neighboring electrodes by performing an EEG propagation speed analysis using emotional stimuli presented using a smartphone. Previous studies have shown that analysis between electrodes was coherence analysis, phase-lock analysis, and phase synchronization analysis, and methodology and clinical cases is almost (Mizuno-Matsumoto et al., 2002, 2005; Cohen et al., 2009; Thatcher, 2012). Additionally, processing information related to word and object encodings has been reported (Stein et al., 1999; Weiss and Rappelsberger, 2000). Propagation speed analysis related to emotion stimuli has not been used in previous studies. The propagation speed analysis was developed based on delay analysis. Govindan et al. (2006) reported that by using propagation speed analysis, researchers could understand the nature of information flow, and ascribe the direction of information flow between centers based on delay analysis. Thus, it can be used to establish the connection and direction between the two signals using delay analysis (Govindan et al., 2005).

### Emotion-related propagation speed changes in alpha activity

Propagation speeds in α frequency were evaluated in relation to the difference between anxiety states of subjects.

In generally, α band activity occurs in an arousal state with resting eye close (Niedermeyer and Silva, 2005). And Knyazev (2007) was reported that α frequency was related to the visual identification function for emotion. However, it was noted that it increased by mental activity, internal activity, and short-term memory such as mental calculation, image, and maintenance of working memory (Kostyunina and Kulikov, 1996; Palva and Palva, 2007). Further, Jensen et al. (2002) were reported that α band activity increased in association with the maintenance of simple memory. In addition, α band activity is reduced by intense mental activity, including anxiety, vigilance, and attention to the stimulus (Avram et al., 2010). Neurotic subjects manifest a low appearance ratio of α frequency, which was associated with anxiety (Markand, 1990). We previously reported that α band activity in the non-stress group was significantly increased during the presentation of unpleasant emotional stimuli, indicating that brain functional activity influences stress resistance (Hayashi et al., 2006).

The results show that propagation speeds for resting and unpleasant sentence stimuli in the inter-hemisphere of the low anxiety group were fast at 0–150 s for both stimuli. However, those of the high anxiety group remained unchanged and showed a low value of propagation speeds.

### Emotion-related propagation speed changes of regional parts

We divided the analysis of propagation speeds under emotional stimuli between neighboring regions into four regions in this study. The results of a Two-Way factorial ANOVA in Figure 7 shows that more significant differences were noted in medial sagittal and medial coronal. In addition, propagation speeds were high for inter-hemisphere (Fp1-Fp2, F3-F4, C3-C4, P3-P4, O1-O2) and both hemispheres of directions (Fp1-F3, Fp2 -F4, F3-C3, F4-C4, C3-P3, P3-P4, P3-O1, P4-O2) in Figures 5, 6. And the direction of propagation speeds at alpha frequency in low anxiety group under unpleasant and unpleasant sentence were from left hemisphere to right hemisphere, that in high anxiety group, by contrast, were from right to left.

The brain inputs emotional visual stimuli from the external word, and performs simple visual processing in the occipital area (Martin et al., 2002). Then, Vision Society of Japan (2001) reported that information is propagated through the cerebral area from the occipital area to the frontal area. Researchers have considered that the emergence of alpha frequency in the occipital region was controlled by a thalamic pacemaker. The thalamic pacemaker is mediated by sagittal nerve fibers that communicate between the frontal and occipital regions in the cerebral hemispheres, and suggested that the α frequency in the occipital region was propagated to the frontal area. Therefore, Okuma (1999) suggested that α frequency emerged from the frontal area. Previous studies have shown a relationship with α frequency and emotional stimuli (Asakawa et al., 2012; Kamo et al., 2013). Prior research indicates that high anxiety subjects manifested reduced information processing at inter-hemispheric region using threatening images tasks (Compton et al., 2008). In addition, Weissman and Banich (1999) hypothesized the callosum as a type of selective filter that can adaptively control information flow between the hemispheres.

The current results of propagation speed may indicate that subjects with high anxiety were not as responsive compared to those with low anxiety. We have suggested that non-properly information processing in the brain was performed by the anxiety state. Additionally, it seems that subjects with high anxiety felt more disgust and anxiety in response to unpleasant stimuli.

These events suggest that information processing in the brain for emotional stimuli differ based on the anxiety state of the subject. In the future, smartphone manufacturers and providers should be educated about the need to develop software and hardware optimal for minimizing anxiety responses.

### Conflict of interest statement

The authors declare that the research was conducted in the absence of any commercial or financial relationships that could be construed as a potential conflict of interest.
